# Sexual Knowledge and Victimization in Adults with Autism Spectrum Disorders

**DOI:** 10.1007/s10803-014-2093-y

**Published:** 2014-03-25

**Authors:** S. M. Brown-Lavoie, M. A. Viecili, J. A. Weiss

**Affiliations:** Department of Psychology, York University, 4700 Keele St., Toronto, ON M3J 1P3 Canada

**Keywords:** Autism spectrum disorders, Sexual knowledge, Sexual victimization, Asperger syndrome, Education

## Abstract

There is a significant gap in understanding the risk of sexual victimization in individuals with autism spectrum disorders (ASD) and the variables that contribute to risk. Age appropriate sexual interest, limited sexual knowledge and experiences, and social deficits, may place adults with ASD at increased risk. Ninety-five adults with ASD and 117 adults without ASD completed questionnaires regarding sexual knowledge sources, actual knowledge, perceived knowledge, and sexual victimization. Individuals with ASD obtained less of their sexual knowledge from social sources, more sexual knowledge from non-social sources, had less perceived and actual knowledge, and experienced more sexual victimization than controls. The increased risk of victimization by individuals with ASD was partially mediated by their actual knowledge. The link between knowledge and victimization has important clinical implications for interventions.

## Introduction

Healthy sexual development is an important achievement for individuals with and without autism spectrum disorders (ASD) alike. Individuals with ASD have been found to display an interest in sexual interactions and to engage in sexual behaviors (Gilmour et al. 2012; Haracopos and Pedersen 2004; Hellemans et al. 2007; Ousley and Mesibov 1991; Van Bourgondien et al. 1997), yet may lack developmentally appropriate education in sexuality, sexual health, and healthy relationships (Hénault 2005). While the relationship between poor sexual knowledge and unsafe sexual practices and sexual victimization is well known in the existing literature, it has rarely been examined with regard to individuals with ASD.

Researchers have found that individuals with ASD have lower levels of sexual knowledge than comparison groups, both when assessed through self-report or parent-report (see Meister et al. 1994; Ruble and Dalrymple 1993; Stokes and Kaur 2005), yet have similar levels of comprehension of sexual language (Gilmour et al. 2012; Hatton and Tector 2010). Parents indicate that their children with ASD tend to have less knowledge of privacy issues related to sexual behavior and receive less information about sexuality from their peers compared to individuals without ASD (Stokes and Kaur 2005; Stokes et al. 2007), and individuals with ASD are less able to define sexual activities than IQ matched peers with developmental delay (Konstantareas and Lunsky 1997).

Researchers have made a distinction between an individual’s actual knowledge and their perception of sexual knowledge (i.e., the amount of accurate knowledge that an individual *believes* they hold), with both reported as important for safe sexual practices (Rock et al. 2005; Ryan et al. 2007). Ryan et al. (2007) found that males are more likely to report higher levels of perceived knowledge than females, and in males, this perceived knowledge is related to condom use, whereas actual knowledge is not (Ryan et al. 2007). In females, perceived knowledge has little impact, with actual knowledge being a more important influence on actual behaviors. It is likely important to take into account the interaction between perception of knowledge and actual knowledge. Having *high perceived* knowledge with *inaccurate actual* knowledge increases the risk for not using contraceptives, compared to individuals with low perceived knowledge (regardless of their actual knowledge) (Rock et al. 2005). To date, no study has explored both actual and perceived sexual knowledge in individuals with ASD, or how these factors may be related to sexual experiences.

Poor sexual knowledge in individuals with ASD may be related to the restricted range of knowledge sources available. Mehzabin and Stokes (2011) found that individuals with ASD were more likely to report television and “by making mistakes” in their list of sexual knowledge sources, and less likely to report social sources. With their inherent sociocommunicative impairments, individuals with ASD likely have fewer opportunities to receive accurate safe sexual health information compared to peers. The difficulties in understanding intentions, missing social subtleties, and limited peer interactions (American Psychiatric Association 2000; Briskman et al. 2001; Frith 1989; Happé et al. 2001), may lead individuals with ASD to miss or misunderstand important sexual knowledge-gaining opportunities and sources (Hénault 2005). In addition, many parents of individuals with ASD report they lack the confidence to discuss sexuality and sexual-related topics with their children for fear that discussing sex will increase adolescents’ sexual interest or behaviors (Konstantareas and Lunsky 1997; Meister et al. 1994).

Most concerning is the potential that the combination of age appropriate sexual motivation and interest, limited sexual knowledge and experiences, and additional social deficits, may place adolescents and young adults with ASD at increased risk for sexual violence victimization. Sexual violence victimization, sexual aggression, and sexual abuse are terms used to label being a victim of the act of coercion, use of drugs or alcohol, or the threat or use of physical force to obtain unwanted sexual contact (Koss and Gidycz 1985). Although some authors have hypothesized links between communication and behavioral deficits inherent to ASD and sexual well-being (Hénault 2005; Sevlever et al. 2013), the literature has yet to adequately examine rates of sexual victimization in adults with ASD. What exists involves documented sexual abuse in children and adolescents. One US community-based study examined rates of sexual abuse in 3,200 children (up to 21 years of age) identified with various disabilities, including autism, from school board administrative databases (Sullivan and Knutson 2000). Children with autism in the education system were not at an increased risk for sexual abuse compared to peers with other disabilities. It was unclear as to whether children with Asperger syndrome were identified in the autism category within the school boards or whether some children were enrolled in the school system without any identification. Subsequent research has found high prevalence rates of sexual maltreatment in youth with ASD, with males being less likely to be sexually abused than females (Mandell et al. 2005). Out of 182 clinical interviews in community service agencies across the United States, Mandell et al. (2005) found that 16.6 % of caregivers reported their child with ASD had experienced sexual abuse. Eighty-six percent of the sample included children with Asperger syndrome. A recent review of the literature indicated that, due to a paucity of research, rates of sexual victimization in this population are unclear for both youth and adults (Sevlever et al. 2013).

The current study explored differences between knowledge sources, actual knowledge, perceived knowledge, and sexual victimization in individuals with high functioning ASD and a comparison group of peers without ASD. It was hypothesized that individuals with ASD would have obtained knowledge from fewer social sources, would have less actual and perceived sexual knowledge, and higher rates of sexual victimization than the comparison group. It was also hypothesized that an increased risk of sexual victimization for individuals with ASD would be mediated by deficits in actual knowledge, perception of knowledge, and social sources of knowledge.

## Methods

### Participants

The current study involved two groups of participants: Ninety-five participants with high functioning ASD between the ages of 19–43 years (*M* = 27.83, *SD* = 4.33) and one hundred and seventeen participants without ASD matched on mean chronological age (18–35 years of age; *M* = 27.60, *SD* = 4.74). Table 1 presents demographic information for both groups. Groups did not significantly differ with respect to the proportion of males versus females (62 % of the ASD group and 56.4 % of the non-ASD group were male), χ^2^ (1, *N* = 211) = .60*, p* = .44. Groups did not differ in visible minority status, education, or in self-reported sexual orientation (heterosexual vs. not heterosexual). The participants with ASD were recruited from community and private agencies that specifically support individuals with ASD. All reported being diagnosed with an ASD and met the clinical cut-off (score of >26) for ASD specified on the Autism Spectrum Quotient (AQ; Baron-Cohen et al. 2001). The AQ has high specificity for discriminating between individuals with ASD and neurotypical individuals (Woodbury-Smith et al. 2005). A number of other studies have used similar methods for support of confirmation of diagnosis (Clopper et al. 2012; Woodbury-Smith et al. 2005). With respect to self-reported diagnosis, 55 % (*n* = 52) of individuals reported that their primary diagnosis was Autism, 41 % (*n* = 39) reported Asperger syndrome, and 4 % (*n* = 4) reported a Pervasive Developmental Disorder. All comparison group participants scored below the ASD cutoff score on the AQ.

**Table 1:** Demographic information and comparisons by group

Demographic variable	ASD *N* (%)	Control *N* (%)	Test for group differences
*Sex*			
Female	36 (38.20)	51 (43.6)	χ^2^ (1, *N* = 211) = .60*, p* = .44
Male	58 (61.70)	66 (56.4)	
*Level of education*			
High school	13 (13.68)	11 (9.4)	χ^2^ (2, *N* = 212) = 1.93, *p* = .38
College	56 (58.94)	65 (55.6)	
University	26 (27.36)	41 (35.0)	
*Visible minority*			
Yes	13 (13.68)	25 (21.4)	χ^2^ (2, *N* = 212) = 2.58, *p* = .28
No	80 (84.21)	88 (75.2)	
Not sure	2 (2.10)	4 (3.2)	
*Sexual orientation*			
Heterosexual	74 (78.71)	75 (67)	χ^2^ (1, *N* = 206) = 3.53, *p* = .06
Not heterosexual	20 (21.28)	37 (33)	

### Materials

All questionnaires and specific questions were selected or adapted in order to ensure they were concrete and could be clearly understood by a grade 8 reading level. All sexual terms were accompanied by definitions, such as “Sexual Intercourse includes anal or vaginal sex (e.g. penis insertion into anal or vaginal openings)”, so as to not presume that participants had prior knowledge of specific sexual acts.

#### Demographics

Participant demographics were obtained across both groups. Variables of interest included age, sex, visible minority status, sexual orientation, level of education, and ASD diagnosis. Among the sexual orientation category participants were asked to indicate if they identified as heterosexual, gay/lesbian, queer, two-spirited, bisexual, or other. Given the low rates of queer, two-spirited, bisexual, and other, the groups were collapsed for any demographic analyses into heterosexual and not heterosexual.

#### Autism Spectrum Quotient (AQ; Baron-Cohen et al. 2001)

The AQ is a 50-item questionnaire designed to measure 5 different areas of functioning related to ASD: social skills, attention switching, attention to detail, communication, and imagination. Participants respond to each statement on a 4-point Likert scale, with answer categories: 1 = *definitely agree*; 2 = *slightly agree*; 3 = *slightly disagree*; 4 = *definitely disagree*. Scoring is dichotomized to indicate presence (score of 1) or absence (score of 0) of the symptom, resulting in scores from 0 to 50. Woodbury-Smith et al. (2005) reported good discriminative validity, high specificity, and good screening properties using a cut-off score of 26 (sensitivity 0.85–0.95, specificity 0.52–0.96), which was used in the current study. In the original validation studies of the AQ two-week test retest reliability scores were reported to be high (*r* = .70; Baron-Cohen et al. 2001). This scale exhibited good internal consistency in the current study (Cronbach’s alpha = .84).

#### Sexual Knowledge Sources

Sexual knowledge sources were examined across three domains: sexually transmitted infections (STIs), sexual behaviors, and contraceptives. Participants indicated if they received their knowledge in each of these domains using a checklist from a total of 11 sources (1 = *yes*; 0 = *no*). The 11 possible sources included 6 social sources and 5 non-social sources, taken from Berten and Van Rossem (2009), Bleakley et al. (2009), and generated by the authors. Social knowledge sources included parents/guardians, teachers, friends/peers, boyfriend/girlfriend/romantic partner, religious figure, and support worker. The non-social sources included television/radio, magazines, Internet, educational brochures and pamphlets, and pornography. A total score for social and non-social sources was created for each of the domains (STIs, sexual behaviors, and contraceptives), ranging from 0 to 6 for social sources and 0 to 5 for non-social sources. Total scores across domains were also calculated (total sources, total social sources, total non-social sources).

#### Sexual Knowledge

The revised Knowledge of Sexual Health questionnaire (Walsh and Ward 2010; Weinstein et al. 2008) is a 37-item questionnaire measuring actual knowledge, knowledge sources, and self-efficacy. The 25 *actual knowledge* questions were used in the current study, related to STIs, contraception, and reproductive health (e.g. “Fertilization of the egg by the sperm (conception) occurs in the woman’s uterus”). Participants respond by indicating if a statement is *True* or *False*. Items are summed with one point for each correct response, with scores ranging 0 to 25. The revised measure has good internal consistency (Cronbach’s alpha = .77), and has been correlated with other sexual knowledge measures (DiClemente et al. 1989; Joseph et al. 1995). This scale also exhibited good internal consistency in the current study for the total score (Cronbach’s alpha = .86).

#### Perceived Knowledge

A 5-item questionnaire created by the authors was used to measure the participants’ perceptions of their sexual knowledge. The questions inquired about perceived knowledge of sexual health, contraception, and risky behavior. The questions were modeled after Ryan and colleagues' (2007) dichotomous one item measure that examined perception of condom knowledge (“You are quite knowledgeable on how to use a condom correctly” True/False). The Appendix includes a copy of the five items. True responses were scored as a 1 and summed, resulting in a score ranging from 0 to 5.

#### Sexual Victimization

The Sexual Experiences Survey, Victimization version was used to examine experiences of sexual victimization (SES; Koss et al. 1987). Modifications to the survey included changing the survey so that both males and females could complete the victimization scales (McConaghy et al. 1993), and rewording of the sexual contact items to make them more explicit in their meaning (Testa et al. 2004). The SES consists of 10 items reflecting varying levels of sexual victimization, on which an individual endorses whether they have experienced that type of victimization. Thus, rather than explicitly asking if they have been victimized (e.g. have you experienced sexual victimization: yes or no), participants were required to indicate if they had experienced a specific situation (e.g. Have you had sexual intercourse when you didn’t want to because someone used their position of authority [boss, teacher, camp counselor, supervisor] to make you?). This method of asking about specific situations is important, as some participants may not realize that their experiences could be classified as victimization (Tourangeay and McNeeley 2003). The items are collapsed into one of four categories based on level of severity (McConaghy et al. 1993). Responses are coded into 0 = *no unwanted sexual experiences* (individual indicates “no” to all 10 items); 1 = *unwanted sexual* *contact* (individual responds “yes” to experiencing one or more of 3 items reflecting contact); 2 = *sexual coercion* (individual responds “yes” to experiencing one or more of 2 items reflecting coercion); 3 = *attempted rape* (individual responds “yes” to experiencing one or more of 2 items reflecting attempted rape); and 4 = *rape* (individual responds “yes” to experiencing one or more of 3 items reflecting rape). The measure has been shown to have good test–retest reliability (Koss and Gidycz 1985), and validity (Koss et al. 1987).

### Procedure

The participants with ASD were recruited through notices regarding the study distributed through community-based programs and organizations offering services to young adults with ASD across Canada and the United States. Notices were also posted on online ASD communities, and distributed by participants to others at their discretion. The comparison group was recruited through Qualtrics’ data system participant database. All participants completed their questionnaires on the online Qualtrics data system (www.qualtrics.com). All participants provided informed consent before accessing the online questionnaire. All participants lived in either the United States or Canada. Participants received a $20 gift card to an online retailer for their participation. The university ethics board approved this research.

In total, 500 people started the survey. Of this total sample 288 were dropped: 50 had <50 % of survey items completed, 56 completed the survey in <10 % of the expected time, 31 completed the survey with an inconsistent response pattern that suggested they were not truthful in their responses (e.g. selected all *strongly agree* throughout the survey despite the counterbalancing of items), and 151 (127 individuals with ASD and 24 comparison group peers) did not meet group criteria (e.g. in the ASD group the AQ scores were below the cut off and in the TD group the AQ scores were above the cut off).

### Data Analysis

Given that sex has been found to impact rates of sexual victimization, sex was entered as control variable in all analyses. A series of 2 (group) × 2 (sex) ANOVAs were calculated to compare sources of sexual knowledge, total perceived, and total actual knowledge between groups. Chi square analyses were calculated to compare the frequency of victimization for sexual contact, sexual coercion, attempted rape, and rape between ASD and non-ASD groups, after examining potential differences based on participant sex. Finally, in order to examine the hypothesis that the increased risk of sexual victimization for individuals with ASD would be mediated by their deficits in actual knowledge, perception of knowledge, and social sources of knowledge, a test of multiple mediation was run using Preacher and Hayes’ (2008) SPSS INDIRECT macro script for testing multiple mediator models with bootstrapping. The INDIRECT macro is most useful in estimating indirect effects when working with smaller sample sizes (e.g. under 400) or if the estimated mediated effect is small or modest (Preacher and Hayes 2004).

## Results

### Sexual Knowledge Sources

In order to test the hypothesis that the participants with high-functioning ASD would have obtained knowledge from fewer social sources, a series of 2 (group) × 2 (sex) ANOVAs were conducted for the total number of sources, total number of social sources, and total number of non-social sources for each domain of sexual knowledge (STIs, sexual behaviors, contraceptives). As shown in Table 2, no differences emerged with respect to sex in any of the analyses. There were also no differences between groups in the total number of sources. Participants with ASD reported obtaining knowledge from *fewer social* sources in each of the three domains: knowledge about STIs, *F*(1, 206) = 11.73, *p* = .001, η_p_^2^ = .05, knowledge regarding sexual behaviors, *F*(1, 206) = 6.95, *p* = .009, η_p_^2^ = .03, and knowledge about contraceptives, *F*(1, 206) = 19.85, *p* < .001, η_p_^2^ = .08. Participants with ASD also reported obtaining knowledge from more *non*-*social* sources in each of the three domains: knowledge about STIs, *F*(1, 206) = 3.76, *p* = .05, η_p_^2^ = .02, knowledge regarding sexual behaviors, *F*(1, 206) = 6.70, *p* = .01, η_p_^2^ = .03, and knowledge about contraceptives, *F*(1, 206) = 5.55, *p* = .02, η_p_^2^ = .03.

**Table 2:** Sources of knowledge by domain and group

Type of knowledge	ASD		Control		*F*-test
	Female *M* (*SD*)	Male *M* (*SD*)	Female *M* (*SD*)	Male *M* (*SD*)	
*Knowledge about STIs*					
Total	3.08 (1.60)	2.41 (1.27)	2.96 (2.03)	2.86 (2.19)	Group: *F*(1, 206) = .39, *p* = .53 Sex: *F*(1, 206) = 2.16, *p* = .14 Interaction: *F*(1, 206) = 1.21, *p* = .27
Social	1.20 (.93)	0.81 (.83)	1.53 (1.15)	1.48 (1.14)	Group: *F*(1,206) = 11.73, *p* = .001 Sex: *F*(1,206) = 2.19, *p* = .14 Interaction: *F*(1, 206) = 1.39, *p* = .24
Non-social	1.88 (1.13)	1.60 (1.10)	1.34 (1.22)	1.37 (1.39)	Group: *F*(1, 206) = 3.76, *p* = .05 Sex: *F*(1, 206) = .91, *p* = .34 Interaction: *F*(1, 206) = 0.43, *p* = .51
*Knowledge about sexual behaviours*					
Total	3.74 (2.08)	3.35 (1.65)	3.51 (2.08)	3.36 (2.44)	Group: *F*(1, 206) = .13, *p* = .72 Sex: *F*(1, 206) = .84, *p* = .36 Interaction: *F*(1, 206) = .18, *p* = .67
Social	1.37 (1.35)	1.10 (1.02)	1.74 (1.04)	1.57 (1.16)	Group: *F*(1, 206) = 6.95, *p* = .009 Sex: *F*(1, 206) = 1.86, *p* = .17 Interaction: *F*(1, 206) = .09, *p* = .76
Non-social	2.37 (1.19)	2.24 (1.20)	1.76 (1.49)	1.78 (1.69)	Group: *F*(1, 206) = 6.70, *p* = .01 Sex: *F*(1, 206) = .07, *p* = .79 Interaction: *F*(1, 206) = .14, *p* = .71
*Knowledge about contraceptives*					
Total	2.97 (1.92)	2.48 (1.44)	3.22 (2.18)	2.86 (2.32)	Group: *F*(1,206) = 1.20, *p* = .27 Sex: *F*(1, 206) = .2.18, *p* = .14 Interaction: *F*(1, 206) = .06, *p* = .81
Social	1.03 (1.22)	0.81 (.98)	1.82 (1.19)	1.50 (1.28)	Group: *F*(1, 206) = 19.85, *p* < .001 Sex: *F*(1, 206) = 2.64, *p* = .10 Interaction: *F*(1, 206) = .10, *p* = .75
Non-social	1.94 (1.16)	1.67 (1.11)	1.39 (1.22)	1.36 (1.51)	Group: *F*(1, 206) = 5.55, *p* = .02 Sex: *F*(1, 206) = .67, *p* = .41 Interaction: *F*(1, 206) = .44, *p* = .51

Chi square tests were then used to compare the frequency of each knowledge source endorsed by individuals with and without ASD. As shown in Table 3, the ASD group was 2.53 to 3.35 times less likely to report obtaining information pertaining to STIs from their parents, teachers, and peers, and 1.66 to 3.51 times more likely to report such knowledge from television/radio and pornography relative to the comparison group. The ASD group was 2.10 to 2.68 times less likely to report obtaining knowledge of sexual behaviors from parents, teachers, and peers, and 1.76 to 4.13 times more likely to report obtaining knowledge about sexual behaviors from support workers, religious figures, educational brochures, the internet and television/radio than the comparison group. Finally, with regards to contraceptives, the ASD group was 2.96 to 4.70 times less likely to report obtaining this knowledge from parents, teachers, and peers compared to the comparison group.

**Table 3:** Breakdown of specific sources of knowledge by domain and group comparisons

Type of knowledge	Knowledge source	ASD *N* (%)	Control *N* (%)	χ^2^	Odds ratios
STI	Parents	11 (11.7 %)	36 (30.7 %)	10.95***	3.35 (CI 1.60–7.04)^a^
	Teachers	31 (33.0 %)	72 (61.5 %)	17.01***	3.25 (CI 1.84–5.74)^a^
	Peers	16 (17 %)	40 (34.1 %)	7.88**	2.53 (CI 1.31–4.50)^a^
	Romantic partner	13 (13.8 %)	16 (13.7 %)	0.01, *ns*	–
	Religious figure	5 (5.3 %)	5 (4.3 %)	0.13 *ns*	–
	Support worker	14 (14.9 %)	7 (6.0 %)	4.62, *ns*	–
	Educational brochures	26 (27.7 %)	42 (35.9 %)	1.62, ns	–
	Television/radio	38 (40.4 %)	24 (20.5 %)	9.96^**^	1.66 (CI 0.86– 3.20)^b^
	Magazines	29 (30.0 %)	33 (28.2 %)	0.18, *ns*	–
	Internet	51 (54.3 %)	59 (50.4 %)	0.31, *ns*	–
	Pornography	15 (16.0 %)	6 (5.1 %)	6.82*	3.51 (CI 1.31–9.45)^b^
Behaviours	Parents	12 (12.8 %)	33 (28.2 %)	7.40**	2.68 (CI 1.30–5.56)^a^
	Teachers	20 (21.3 %)	48 (41.0 %)	9.31**	2.23 (CI 1.19–4.14)^a^
	Peers	30 (31.9 %)	58 (49.6 %)	86.68**	2.10 (CI 1.193.69)^a^
	Romantic partner	27 (28.7 %)	45 (38.5 %)	2.20, *ns*	–
	Religious figure	12 (12.8 %)	5 (4.3 %)	5.07*	3.28 (CI 1.11–9.67)^b^
	Support worker	12 (12.8 %)	4 (3.4 %)	6.50*	4.13 (CI 1.23–13.28)^b^
	Educational brochures	38 (40.4 %)	30 (25.6 %)	5.22*	1.97 (CI 1.10–3.53)^b^
	Television/radio	48 (51.1 %)	28 (30.8 %)	8.60**	2.35 (CI 1.34–4.13)^b^
	Magazines	35 (37.2 %)	41(35.0 %)	0.11, *ns*	–
	Internet	66 (70.2 %)	67 (57.3 %)	3.75*	1.76 (CI 0.99–3.13)^b^
	Pornography	26 (27.7 %)	34 (29.1 %)	0.05, *ns*	–
Contraceptives	Parents	10 (10.6 %)	42 (35.9 %)	17.91***	4.70 (CI 2.21–10.03)^a^
	Teachers	24 (25.5 %)	65 (55.5 %)	19.25***	3.65 (CI 2.02–6.57)^a^
	Peers	20 (21.3 %)	52 (44.4 %)	12.45***	2.96 (CI 1.65–5.47)^a^
	Romantic partner	13 (13.8 %)	26 (22.2 %)	2.43, *ns*	–
	Religious figure	5 (5.3 %)	3 (2.5 %)	1.09, *ns*	–
	Support worker	12 (12.8 %)	4 (3.4 %)	6.50, *ns*	–
	Educational brochures	38 (40.4 %)	46 (39.31 %)	0.01, *ns*	–
	Television/radio	28 (30.1 %)	22 (18.8 %)	3.65, *ns*	–
	Magazines	34 (36.2 %)	30 (25.64 %)	2.73, *ns*	–
	Internet	54 (57.4 %)	54 (46.1 %)	2.66, *ns*	–
	Pornography	11 (11.7 %)	9 (7.7 %)	0.98, *ns*	–

*Note* All CI are 95 % confidence intervals of the odds ratios

All odds ratios are calculated in favor of the group with the greatest number of participants reporting they obtained knowledge from the source being measured

* *p* ≤ .05; ** *p* ≤ .01; *** *p* ≤ .001

^a^ASD group less likely than the comparison group

^b^ASD group more likely than the comparison group

### Actual and Perceived Knowledge

A similar 2 (group) × 2 (sex) ANOVA was calculated to test the hypothesis that individuals with ASD would have less perceived knowledge than the comparison group. There was a significant main effect for group *F*(1, 206) = 6.80, *p* = .01, η_p_^2^ = .03, with participants with ASD (*M* = 4.23, *SD* = 1.01) reporting less perceived knowledge than the comparison group (*M* = 4.58, *SD* = .71). There was no main effect for sex *F*(1, 206) = 2.46, *p* = .12, and no significant interaction, *F*(1, 206) = .23, *p* = .63.

With regard to actual knowledge, there was a significant main effect for group *F*(1, 206) = 60.58, *p* < .001, η_p_^2^ = .23, with participants with ASD (*M* = 17.44, *SD* = 6.27) having less actual knowledge than the comparison group (*M* = 23.54, *SD* = 5.51). There was also a significant main effect for sex, *F*(1, 206) = 16.23, *p* < .001, η_p_^2^ = .07, with women (*M* = 22.95, *SD* = 6.65) having higher actual knowledge than men (*M* = 19.34, *SD* = 6.05). There was no sex by group interaction, *F*(1, 206) = 1.89, *p* = .17. Given the sex main effect, subsequent independent samples *t* tests confirmed that men with ASD (*M* = 16.63, *SD* = 5.85) reported significantly less knowledge than men without ASD (*M* = 21.68, *SD* = 5.22), *t*(121) = −5.06, *p* < .001, and women with ASD (*M* = 18.72, *SD* = 6.78) reported significantly less knowledge than women without ASD (*M* = 25.94, S*D* = 4.68), *t*(85) = −5.87, *p* < .001.

### Sexual Victimization

Chi square analyses and odds ratios were used to examine the hypothesis that individuals with ASD would be more likely to experience various forms of sexual victimization than the comparison group. As seen in Table 4, individuals with ASD were almost 3 times more likely to experience unwanted sexual contact, 2.7 times more likely to experience sexual coercion, and 2.4 times more likely to experience rape than the comparison group. There were no significant differences between groups in terms of attempted rape. Seventy-eight percent of respondents with ASD reported at least one occurrence of sexual victimization, compared to 47.4 % of the comparison group. Males with ASD were more likely to experience victimization compared to males without ASD, for contact, coercion, and rape. Women with ASD were more likely to experience sexual contact and sexual coercion than women without ASD. There were no significant differences between men and women within the ASD group or within the control group for any types of victimization (all *p* > .05).

**Table 4:** Group comparisons of rates of sexual victimization

	ASD *N* (%)	CONTROL *N* (%)	χ^2^	Odds ratios
Sexual contact*	65 (70 %)	51 (44 %)	14.50***	3 (CI 1.7–5.3)
Sexual coercion*	36 (39 %)	22 (19 %)	10.14***	2.7 (CI 1.5–5.1)
Attempted rape	25 (27 %)	24 (20.5 %)	1.18, *ns*	–
Rape*	29 (31.5 %)	19 (16.4 %)	6.63**	2.4 (CI 1.2–4.5)

All odds ratios are calculated in favor of the group with the greatest number of participants reporting each type of victimization. All comparisons were calculated in favor of the ASD group

* *p* ≤ .05; ** *p* ≤ .01; *** *p* ≤ .001

### Relationship Between Knowledge, Social Sources, and Victimization

Finally we estimated the extent to which ASD status represented a risk factor for sexual victimization after taking into account the marked between group differences in perceived knowledge, actual knowledge, and sexual sources. We used multiple mediation, with perceived knowledge, actual knowledge, and total sources as potential mediators on the impact of ASD status (having an ASD vs. not) on predicting victimization (any experienced victimization vs. none). The multiple mediation analysis was run using an SPSS supplemental macro script for testing multiple mediator models with bootstrapping (see Preacher and Hayes 2008). Sex was entered as a control variable. Table 4 shows the unstandardized coefficients of each pathway, and the bootstrapping results based on 1,000 resamples.

**Fig. 1:**
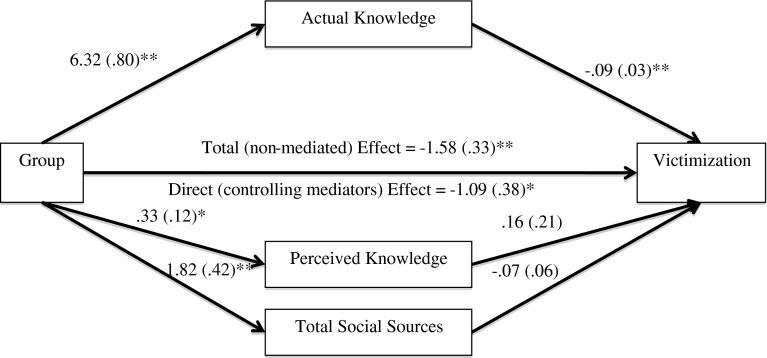
Model of relationship among group, actual knowledge, perceived knowledge, total social sources, and victimization. *Note* Values presented are coefficients and standard errors after controlling for sex. * *p* ≤ .05; ** *p* ≤ .001

As shown in Fig. 1 and Table 5, the total direct effect (path c) of ASD status was a significant predictor of victimization, before entering the mediator variables, *z* = −4.79, *p* < .001. The direction of estimates in the mediator pathways (path a) indicated that having an ASD was associated with less perceived knowledge, *t* = 2.79, *p* = .005, less actual knowledge, *t* = 7.87, *p* < .001, and fewer social sources, *t* = 4.32, *p* < .001. The total indirect effect from bootstrapping suggested the presence of mediation, CI −1.22 to −0.19, the result of the fact that less actual knowledge was associated with more victimization (path b), *t* = −2.98, *p* = .003. Social sources and perceived knowledge were not significant mediators in the overall model. After entering the mediators and the control variable, ASD status remained significant, *z* = −2.90, *p* = .004, suggesting that actual knowledge was a partial mediator.

**Table 5:** Multiple mediation analysis results for the mediating effect of perceived, actual, and sources of knowledge on the relationship between group and sexual victimization after controlling for sex

DV	IV, mediators, and control	Path	B	SE	z/t	*p*	Bootstrapping for indirect results		
							Point estimate	95 % CI	
								Lower	Upper
Victimization	Sex	Control	−1.10	.42	−2.61	.009			
	Group	C	−1.75	.38	−4.58	<.001	−.78	−1.43	−.23
		C′	−1.24	.43	−2.85	.004			
	Perceived knowledge	A	.30	.14	2.21	.029	.09	−.02	.30
		B	.31	.23	1.38	.170			
	Actual knowledge	A	6.66	.89	7.45	<.001	−.80	−1.50	−.23
		B	−.12	.04	−3.22	.001			
	Total number of social sources	A	1.81	.46	3.95	<.001	−.07	−.38	.17
		B	−.04	.06	−.63	.525			

## Discussion

The goals of the current study were to examine sexual knowledge and risk of victimization in adults with high functioning ASD. We found that individuals with ASD obtain less of their sexual knowledge from social sources and more from non-social sources, have less perceived and actual knowledge, and experience more sexual victimization than individuals without ASD. We also found that the increased risk of victimization by individuals with ASD is partially mediated by their level of actual knowledge.

### Social Sources

Our finding that the individuals with ASD obtain less of their knowledge from social sources is in line with previous research. Mehzabin and Stokes (2011) also found important differences between individuals with ASD and typically developing peers’ self-cited sources of sexual education. While typically developing individuals indicated sources such as peers, parents, and teachers, individuals with ASD included television and “by making mistakes” in their list. In the current study, we found that individuals with ASD were less likely to report receiving knowledge from peers, parents, and teachers across all types of sexual knowledge. Core social deficits associated with ASD, and the fact that individuals with ASD face rejection and social isolation, may interfere with important opportunities for gaining sexual knowledge from these social sources (Ousley and Mesibov 1991). Difficulties in understanding intentions, missing social subtleties, and limited peer interactions may lead individuals with ASD to be more likely to miss or misunderstand important sexual knowledge-gaining opportunities (Hénault 2005). In addition, many parents of individuals with ASD lack confidence in discussing sexual-related topics for fear that discussing sex will increase adolescents’ sexual interest or behaviors (Konstantareas and Lunsky 1997; Meister et al. 1994). The reported lack of knowledge from teachers is also concerning. While little research has been done to examine the sexual health curriculum in schools for individuals with ASD, it may be that individuals with ASD are being removed for sexual health classes for various reasons or that the sexual curriculum may not be adapted to their level of understanding (Hénault 2005). The lack of these social sources means that individuals with ASD are left to obtain the majority of their sexual knowledge from non-social and often unmonitored sources, and in the current study, were more likely than peers to obtain knowledge from television/radio, pornography, and the internet (depending on the type of sexual knowledge). Some of these non-social sources have been found to lead to inaccurate sexual knowledge (Berten and Van Rossem 2009). These findings are important given that the development of accurate sexual knowledge has been found to be important for safe sexual practices (Aspy et al. 2007; Holtzman and Rubinson 1995; Hutchinson et al. 2003; Whitaker and Miller 2000; Whitaker et al. 1999).

### Actual and Perceived Knowledge

Previous research examining the levels of perceived and actual knowledge is relatively understudied within the ASD population. Our finding that individuals with ASD have lower levels of perceived knowledge is particularly novel as we know of no previous studies where self-reports of perceptions have been examined. Our finding that individuals with ASD have lower levels of actual knowledge is consistent with findings from parent report studies (Meister et al. 1994; Ruble and Dalrymple 1993) and self-report studies (e.g. knowledge of privacy issues, see Mehzabin and Stokes 2011; Stokes and Kaur 2005). These results indicate that individuals with ASD are lacking important sexual knowledge related to protecting themselves against STIs, sexually appropriate behaviors, and safe sexual practices. Having an understanding of risk factors, how to protect against infection and risky situations, and appropriate behaviors are important steps in being able to reduce the risk of victimization.

### Sexual Victimization

The high rates of sexual victimization found in the current study is of tremendous concern. Seventy-eight percent of respondents with ASD reported at least one occurrence of sexual victimization, compared to 47.4 % of the comparison group. While other authors have noted that individuals with ASD are more vulnerable to social victimization, such as bullying (e.g. Shtayermman 2007), this is the first study utilizing self-report to highlight the critical potential risk of sexual victimization. Individuals with ASD were between two and three times more likely to experience sexual contact victimization, sexual coercion victimization, and rape than comparison group. Importantly, individuals with ASD were found to be at a higher risk of sexual victimization regardless of sex, with males with ASD being more likely to be victimized compared to males without ASD, and females with ASD being more likely to be victimized compared to females without ASD.

The current study used multiple mediation analysis to further explain the higher rate of victimization found in the ASD group relative to the comparison group. Understanding *why* individuals with ASD are at an increased risk through mediation analysis is important to begin to develop ways of addressing the problem. Although sources of knowledge and perceived knowledge were related to ASD status, only the level of actual knowledge mediated the association of ASD and victimization. Having an ASD is indeed related to more experiences of sexual victimization, and lower levels of actual knowledge can explain part of this relationship. Interventions aimed at increasing actual knowledge may be an important preventative tool for individuals with ASD. Currently few programs or guidelines exist that provide guidance for sexuality education in this population (Hénault 2005; Visser et al. 2013; Travers and Tincani 2010). Determining the best sources of information, of program delivery, and ways to tailor content to developmental and cognitive levels may assist in the prevention of sexual violence victimization experiences in this population. Schools, legislators, parents, researchers, and individuals with ASD can work together to determine best practice methods for teaching individuals with ASD and for reducing the stigmatization associated with sexuality and ASD.

Actual knowledge was only a partial mediator, and future research is needed to examine what other factors may further explain the relationship between ASD and sexual victimization, in order to develop more targeted interventions. For example, the sociocommunicative and behavioral deficits of individuals with ASD may impact safety and sexual well-being in interpersonal interactions (Byers et al. 2013; Sevlever et al. 2013). More specifically, individuals with ASD may have difficulty (a) determining “safe” and “unsafe” behaviors, social contexts, and sexual experiences, (b) understanding others’ negative intentions and (c) asserting their own will and wishes with respects to sexual interactions (Ballan 2012; Sevlever et al. 2013).

Our study found increased rates of victimization in both the ASD and in the comparison group relative to previous studies of typically developing individuals (Brennan and Taylor-Butts 2008; Testa et al. 2004; Tjaden and Thoennes 2000). For example, Testa et al. (2004) found that 37.8 % of women in the general population reported some type of victimization using the SES. Brennan and Taylor-Butts (2008) examined police data of reported crimes in Canada and found rates of sexual victimization across the lifespan to be 3,248 incidents per 100,000 in females versus 664 incidents per 100,000 in males. The increased rates of reported victimization in the current study are may be due to a number of methodological differences in the current study that allowed people to identify and feel comfortable disclosing sexual victimization. Specifically, participants were asked to rate if they had experienced specific, concrete, and explicit situations (e.g. “Have you given into sex play (fondling, kissing, or petting, but not intercourse) when you didn’t want to because you were overwhelmed by someone’s continual arguments and pressure?”) rather than simply being asked if they had been sexually victimized. This may have increased reporting, as participants may have not recognized that these experiences were examples of sexual victimization, as various other studies rely on solely on asking about victimization without examples or examine crime report data (Brennan and Taylor-Butts 2008). As well, the increased anonymity that is inherent in self-administered computerized questionnaires has been found to lead to increase rates of reporting risky behaviors, such as sexual behavior and drug use (Turner et al. 1998), as the participants are not as likely to experience response bias, and the current study employed such a design, compared to previous paper-and-pencil surveys where reporting was not anonymous (e.g. Koss and Gidycz 1985; Testa et al. 2004). Research has found that men and women both indicate shame, guilt and embarrassment as well as confidentiality concerns as barriers to reporting sexual victimization (Sable et al. 2006), and this contributes to sexual victimization being less likely to be reported to the police than on victimization surveys (Brennan and Taylor-Butts 2008).

This is the first study to use self-report to ask adults with ASD about their actual and perceived sexual knowledge and experiences of victimization. As indicated with other special populations, self-reported health and life experiences are important for the evaluation of current well-being and health care, yet are often ignored in favor parent or caregiver reporting (Fujiura 2012; McVilly et al. 2000). In the United States, self-report has been proposed as one of 20 core indicators of quality of care and national health (Institute of Medicine 2009), and in health research, self-report has been suggested as crucial for gaining an understanding of personal experiences and more accurately capturing an integrative picture of an individuals biopsychosocial well being (Albrecht 1996; Fujiura 2012). This is particularly the case for experiences of victimization, which may not be fully known by caregivers (e.g. Rønning et al. 2009).

This study has a number of limitations. It was limited to convenience sampling, and the self-selection may limit generalizability to the larger group of adults with ASD. The participants’ diagnoses were confirmed with self-report and the AQ. While this method of identification has been used previously, we cannot absolutely verify the ASD diagnosis. Future research in this domain would benefit from more stringent methods of confirmation of diagnosis by independent clinicians. The data collected pertained specifically to sexual victimization occurring after 14 years of age, which may have limited the rates of victimization reported. The methodology was cross-sectional and correlational in nature, and a longitudinal design examining how actual and perceived knowledge predict later victimization is needed to speak further to causality and directionality of effects. Further, research has found associations between victimization in childhood and later victimization in adolescence and adulthood, and this association has yet to be examined in the ASD population (Humphrey and White 2000).

## Conclusion

This study contributes to the literature by providing initial evidence for a link between sexual victimization and a lack of sexual knowledge in an ASD sample. Having low levels of sexual knowledge places individuals at risk above and beyond what can be accounted for by having an ASD alone. The link between knowledge and victimization has important clinical implications and the high rates of victimization found in the current study is a cause for concern. Given the negative impact that sexual victimization can have on an individual’s mental health and well-being, research is needed to determine best practices for proactive prevention, through teaching of sexually related information to children, adolescents, and adults with ASD. Further research is needed to examine the association between childhood victimization and adult victimization in this population as well as other individual and contextual factors that contribute to this increased risk for victimization.

## Appendix: Perceived Knowledge Questionnaire

Please answer the following questions regardless of your sexual experience (answer even if you have not had sex).

**Table:**

	Response	
1. I know how to put a condom on correctly	True	False
2. I know how to use other contraceptive methods (e.g. oral birth control, vaginal rings, etc.)	True	False
3. I know what sexually transmitted infections (STI/STDs) are	True	False
4. I know how to protect myself against (STI/STDs)	True	False
5. I know how to decrease the likelihood of pregnancy when sexually active	True	False
